# Welches Präventionspotential bietet die hausärztliche Praxis aus Sicht pflegender Angehöriger? – Befunde einer qualitativen Interviewstudie

**DOI:** 10.1007/s10354-021-00880-4

**Published:** 2021-09-16

**Authors:** Julian Wangler, Michael Jansky

**Affiliations:** Zentrum für Allgemeinmedizin und Geriatrie, Universitätsmedizin Mainz, Am Pulverturm 13, 55131 Mainz, Deutschland

**Keywords:** Pflegende Angehörige, Hausarzt, Identifizierung, Belastungen, Bedürfnisse, Betreuung, Caregivers, General practitioner, Identification, Strain, Needs, Care, Support

## Abstract

Die Unterstützung von pflege- und hilfsbedürftigen Personen wird oft durch pflegende Angehörige übernommen. Bei der Betreuung dieser Zielgruppe können Hausärzt*innen eine bedeutende Rolle einnehmen, wenn sie sich auf die Problematiken und Wünsche Pflegender einstellen.

Ziel der explorativen Studie ist es, hausärztliche Betreuungsbedürfnisse mit tatsächlich erlebter Betreuung zu kontrastieren und dadurch Ansätze für eine Stärkung der hausärztlichen Rolle im Themenzusammenhang herauszuarbeiten.

Über 13 Online-Pflegeforen wurden insgesamt 37 pflegende Angehörige rekrutiert, mit denen zwischen September 2020 und März 2021 telefonische Interviews geführt wurden.

Die Mehrheit der Interviewten erachtet Hausärzt*innen als wichtige Unterstützungsinstanz mit hoher Kompetenz- und Vertrauenszuweisung. Geschätzt wird die hausärztliche Kenntnis der persönlichen Betreuungssituation, die Ansprechbarkeit bei verschiedensten Problemlagen und die Hinwendung zum Pflegebedürftigen. Allerdings fällt auf, dass die Kommunikation über die Pflege oft erst deutlich verzögert erfolgt (verspätete Identifizierung und Ansprache Pflegender). Auch nehmen Hausärzt*innen nicht immer im selben Maße Rücksicht auf die Bedürfnisse von Angehörigen wie sie auf Gepflegte eingehen. Nur ein Teil der Ärzt*innen verweist Angehörige zu Beratungs- und Hilfsangeboten.

Die Hausarztpraxis kann eine zentrale Rolle bei der Unterstützung pflegender Angehöriger spielen. Hierfür ist eine frühzeitige Erkennung und Einbeziehung von pflegenden Angehörigen unverzichtbar. Zur effektiven Unterstützung einer gelingenden Pflege ist es wichtig, die Bedürfnisse, Wünsche und Belastungen Pflegender und Gepflegter gleichermaßen zu berücksichtigen. Konsequente Verweise auf Hilfsangebote erleichtern pflegenden Angehörigen die Organisation der Pflege und gewähren (psychosoziale) Unterstützung.

**Zusatzmaterial online:**

Zusätzliche Informationen sind in der Online-Version dieses Artikels (10.1007/s10354-021-00880-4) enthalten.

## Abstract (en)

The support of people in need of care and assistance is often provided by caring relatives. General practitioners can play an important role in supporting this group, if they adapt to the problems and wishes of family caregivers.

The aim of the exploratory study is to contrast care needs of family caregivers regarding the GP support with actually experienced care and, thereby, work out approaches for strengthening the GP’s role.

A total of 37 family caregivers were recruited via 13 internet forums focused on caregiving. Telephone interviews were conducted between September 2020 and March 2021.

The majority of those interviewed consider the GP to be an important support body with a high level of competence and trust. The interviewees praise the GP’s knowledge of the personal care situation, the responsiveness to a wide variety of problems and the focus on those in need of care. However, communication about the caring activity is often delayed significantly (late identification and addressing of family caregivers). GPs do not always address the needs of relatives to the same extent as they do to those in need of care. Only some of the doctors refer caregivers to offers of help and support.

GPs can play a central role in supporting family caregivers. An crucial prerequisite for this is that family caregivers are recognized and involved at an early stage. In addition, it is important that GPs take into account the needs, desires and stresses of both caregivers and those being cared for. Consistent references to offers of help make it easier for family caregivers to organize care and to receive (psychosocial) support.

In der EU-27 sind heute über 20 % der Bevölkerung 65 Jahre oder älter [1, 2]. Daraus ergibt sich ein wachsender Bedarf nach Pflege und Betreuung. Für Deutschland zeigt sich dieser Bedarf anhand von ca. 4,1 Mio. als formell pflegebedürftig eingestuften Menschen [3]. Rechnet man informell-unentgeltliche Unterstützungs- und Pflegetätigkeiten hinzu, erhöht sich diese Zahl auf ca. 5,5 Mio. [4, 5].

Informelle Pflege wird überwiegend im häuslichen Umfeld durch private Pflegepersonen erbracht, die bei der Betreuung und Versorgung von ihnen nahestehenden hilfsbedürftigen Menschen einen beträchtlichen Teil der Fürsorge tragen [6–8]. Repräsentativdaten zufolge unterstützen mehr als 16 % der 40- bis 85-Jährigen mindestens eine Person regelmäßig bei der Bewältigung des Alltags [9]. Hiervon leistet gut jeder Dritte Pflege im engeren Sinne, wobei die Hauptlast meist von weiblichen Familienmitgliedern getragen wird [9, 10].

Obwohl Untersuchungen nachweisen konnten, dass eine pflegende Tätigkeit mit subjektivem Sinnerleben einhergehen kann [11, 12], ist sie aufgrund der physischen und mentalen Belastung mit einem höheren Gesundheitsrisiko assoziiert [10, 13–15]. Entsprechend verbreitet sind unter Pflegenden Beschwerden wie Erschöpfungszustände und depressive Symptome [8, 16–18]. Hat eine Auseinandersetzung mit den Folgen der Erkrankung im Vorfeld nicht stattgefunden und wurden Vorsorgemaßnahmen nicht ergriffen, kommt es nicht selten zu Überforderungssituationen [15].

Um solche Krisen zu vermeiden und die Resilienzfähigkeit Pflegender zu fördern, sind diverse Unterstützungsangebote etabliert worden. In Deutschland zählt hierzu die Tätigkeit von Pflegestützpunkten, ambulanten psychiatrischen Diensten und Demenz-Netzwerken. Allerdings zeigen Arbeiten, dass solche Angebote nur von einem Teil pflegender Personen genutzt werden [19, 20].

Aufgrund der kontinuierlichen Betreuung und langjähriger Kenntnis gelten Hausärzt*innen als gut geeignet, um häusliche Pflegesettings zu begleiten und gezielt auf pflegende Angehörige einzugehen [6, 21–23]. Abgesehen von der Diagnostik und Therapie gesundheitlicher Beschwerden können Hausärzt*innen im Gespräch mit Pflegenden informierend und beratend tätig werden, psychosoziale und emotionale Unterstützung gewähren und sich ein Bild von den Betreuungsbedingungen verschaffen, sodass Bedarfe rechtzeitig adressiert werden können. Indem Hausärzt*innen zu Hilfs- und Beratungsangebote verweisen, können sie die Weichen für eine längerfristig gelingende Pflege stellen und Pflegenden durch Kenntnis geeigneter Ansprechpartner Kompensations- und Entlastungsmöglichkeiten aufzeigen [19, 24].

Da Übergänge hin zu informellen Pflegepersonen oft fließend sind, kann es für das hausärztliche Team herausfordernd sein, pflegende Angehörige frühzeitig zu identifizieren [7, 22]. Schwierigkeiten ergeben sich, wenn die gepflegte Person nicht von der Praxis versorgt wird, in der der Angehörige selbst Patient ist [10]. Gerade in solchen Fällen kann es passieren, dass Allgemeinärzt*innen vorrangig die gepflegte Person wahrnehmen [25, 26] und Belastungen Angehöriger nicht ausreichend im Blick haben [21].

Eine Befragung der Kassenärztlichen Bundesvereinigung kommt zu dem Ergebnis, dass ca. 60 % der pflegenden Angehörigen mit ihrem Hausarzt über ihr Pflegeengagement sprechen [23]. Mit Blick auf den Grad und die Art der hausärztlichen Unterstützung sowie die Betreuungsbedürfnisse Pflegender mangelt es gerade für den deutschsprachigen Raum an belastbaren Studien. Eine im Jahr 2020 durchgeführte Online-Befragung von insgesamt 612 pflegenden Personen, die in 17 Internetforen zum Thema Pflege geschaltet war [27], hat gezeigt, dass eine deutliche Mehrheit der Befragten die hausärztliche Kenntnis der Betreuungssituation, die Ansprechbarkeit bei pflegebezogenen Problemlagen und die Hinwendung zur Pflegeperson positiv beurteilt. Rund die Hälfte gibt an, Verweise auf Beratungs- und Hilfsangebote über den Hausarzt erhalten zu haben; ein ähnlich hoher Anteil bekundet, sich als pflegende Person rechtzeitig vom Hausarzt erkannt und einbezogen gefühlt zu haben. Die Ergebnisse einer Regressionsanalyse zeigen, dass die genannten Aspekte als Einflussfaktoren für die subjektiv erlebte Zufriedenheit mit der hausärztlichen Unterstützung sowie das Gefühl, die Pflegesituation bewältigen zu können, bedeutsam sind.

## Erkenntnisinteresse

Die vorliegende Studie wurde explorativ ausgestaltet und soll Bedürfnisse, Wünsche und Erfahrungen pflegender Angehöriger in Bezug auf die hausärztliche Unterstützung beleuchten. Das Erkenntnisinteresse bündelt sich in folgenden Fragestellungen:

- Welche Bedeutung hat die Unterstützung durch Hausärzt*innen für pflegende Angehörige?
- Was sind vorrangige Betreuungsbedürfnisse und -wünsche pflegender Angehöriger?
- Inwiefern sprechen pflegende Angehörige mit dem Hausarzt über ihre pflegende Tätigkeit?
- Wie lassen sich die mit der hausärztlichen Versorgung in Bezug auf die artikulierten Bedarfe gemachten Erfahrungen beschreiben?
- Welche Verbesserungsansätze lassen sich für das hausärztliche Setting ableiten?

## Material und Methoden

### Studiendesign und Setting

Die Studie steht im weiteren Kontext eines Innovationsfonds-Modellprojektes zur ambulanten medizinisch-pflegerischen Demenzversorgung (*DemStepCare*). Dabei steht die Optimierung der hausarztbasierten Demenzversorgung im Zentrum.

Der Studie ging die angesprochene Online-Befragung pflegender Angehöriger [27] voraus, die in der Breite Betreuungsbedürfnisse und Erfahrungen in Bezug auf die hausärztliche Versorgung ermitteln sollte. Die vorliegende qualitative Arbeit baut entsprechend auf den Resultaten der quantitativen Vorstudie auf mit dem Ziel, zu prüfen, inwieweit sich die Ergebnisse bestätigen bzw. ergänzen lassen.

Das Haupterkenntnisinteresse lag dabei auf einer erklärenden Vertiefung von Erkenntnissen. Die quantitative Befragung erbrachte z. B. Hinweise darauf, dass nicht alle Bedürfnisse pflegender Angehöriger im hausärztlichen Praxisalltag abgedeckt werden. Eine qualitative Studie bietet die Möglichkeit, gezielt auf bestimmte Aspekte und Zusammenhänge einzugehen, Nachfragen zu stellen und mögliche Problemlagen mithilfe der subjektiven Sicht und Erfahrung von pflegenden Personen genauer zu erfassen.

### Leitfaden

Da es sich um eine mehrteilige Studienreihe handelt, wurde bei der Erstellung des Leitfadens auf die vorangegangene quantitative Arbeit zurückgegriffen. Ergo orientierte sich die Konzeption des Instruments eng am Fragebogen [27], was auch mit dem Ziel korrespondiert, ergänzende bzw. vertiefende Einblicke in die Wahrnehmung pflegender Angehöriger mit Blick auf die untersuchte Thematik zu beziehen. Teilweise wurden gestützte, d. h. mit Antwortvorgaben abgefragten Fragen in offene Fragen umgewandelt, um eine unvoreingenommene Exploration zu ermöglichen. Dies betraf insbesondere Fragen zur Bedeutung des hausärztlichen Settings für die eigene Pflegetätigkeit sowie die Gegenüberstellung von artikulierten Bedürfnissen und real geleisteter hausärztlicher Betreuung.

Über die enge Orientierung an der erwähnten Online-Befragung hinaus wurde bei der Entwicklung bzw. Adjustierung des semistrukturierten Leitfadens zusätzlich auf eine allgemeine Literaturrecherche zurückgegriffen. Dabei wurden solche Arbeiten fokussiert, in denen pflegende Angehörige und ihre (potenzielle) Unterstützung im hausarztbasierten Setting im Zentrum der Betrachtung stehen [12, 17, 19, 22, 24, 26], u. a. die Arbeiten von Geschke et al. [19], Greenwood et al. [22, 26] und Joling et al. [17].

Der Fokus des letztendlichen Leitfadens lag auf folgenden Dimensionen: Bedeutung des hausärztlichen Settings für die Unterstützung der eigenen Pflegetätigkeit; Zustandekommen und Häufigkeit des Gesprächs über die Pflegetätigkeit; Erwartungen und Bedürfnisse in Bezug auf die hausärztliche Unterstützung; tatsächlich erlebte Unterstützung und deren Beurteilung; Wünschen und Anregungen zur Verbesserung der hausärztlichen Betreuung (s. Anhang).

Aufgrund der quantitativen Vorstudie, die einen umfassenden Vortest des verwendeten Instruments beinhaltete, wurde darauf verzichtet, den Leitfaden einem separaten Pretest zu unterziehen. Die interne Konsistenz für die Online-Befragung war für sämtliche verwendeten Skalen berechnet worden [27]. Diese kann insbesondere für die Itemsets im Zentrum der Studie als gut bezeichnet werden (Cronbachs α = 0,891 bzw. 0,832).

### Rekrutierung

Zielgruppe der Studie waren alle Arten pflegender Angehöriger. Die Teilnehmer*innen wurden über Diskussionsforen im Internet rekrutiert, die sich auch oder schwerpunktmäßig an pflegende Angehörige richten.

Die Auswahl der Foren erfolgte bereits für die Online-Befragung; dies geschah größtenteils über eine Suchmaschinenrecherche, bei der Schlagwörter wie „Pflegende“, „Pflegende Angehörige“ und „Pflegepersonen“ verwendet wurden (insgesamt wurden 29 Foren recherchiert). Anschließend wurde mit den Administratoren Kontakt aufgenommen und um eine formelle Erlaubnis gebeten, über das jeweilige Forum Befragungsteilnehmer*innen rekrutieren zu dürfen. Es war ohne Aufwand machbar, die 17 Foren, die bereits zur Durchführung der Vorstudie ihr Einverständnis gegeben hatten, für die vertiefende qualitative Studie zu gewinnen. Auf Grundlage der für jedes Forum einsehbaren registrierten Mitgliederzahl gehen die Autoren davon aus, dass die Foren in Summe theoretisch bis zu 11.000 pflegende Angehörige erreichen.

Im Anschluss an die Gewährung der Durchführung wurde ab August 2020 ein Aufruf in Form eines Thementhreads gestartet, bei dem über das allgemeine Thema Auskunft gegeben wurde. Personen, die bereit waren, für ein Interview zur Verfügung zu stehen (keine Incentives), konnten sich bis Jahresende 2020 über eine hinterlegte Emailadresse melden. Nach freiwilliger Meldung (insgesamt 37 Personen aus 13 der 17 Foren) wurden vorab mehrere allgemeine Merkmale erhoben (Geschlecht, Alter, Bildungsabschluss, Berufstätigkeit, allgemeine Wohnumgebung, Dauer der Pflegetätigkeit). Einschlusskriterium war, dass seit mindestens sechs Monaten eine pflegende, unterstützende oder betreuende Tätigkeit für eine nahe stehende Person erbracht wird.

Letztlich wurden mit allen der 37 Interessent*innen Interviews durchgeführt. Zwei der Interviewten hatten bereits an der Online-Befragung teilgenommen.

### Durchführung

Alle Interviews wurden im Wechsel von den Autoren, bei denen es sich um zwei Wissenschaftler im Bereich der allgemeinmedizinischen Versorgungsforschung handelt, im Zeitraum zwischen September 2020 und März 2021 auf telefonischem Wege durchgeführt. Die Dauer der Interviews betrug zwischen 25 und 65 min.

Im Vorfeld erhielten die Interviewten eine Aufklärung über das Gesprächsthema sowie eine Einverständniserklärung. Diese beinhaltete die Zusicherung einer strikten Pseudonymisierung sowie einer Löschung der Gesprächsaufzeichnungen nach Abschluss der Auswertung.

### Auswertung

In Folge der Transkription wurde die Auswertung der Interviews auf Grundlage der qualitativen Inhaltsanalyse nach Mayring [28] im Team mithilfe der Software MAXQDA durchgeführt. Als Vorbereitung wurden die verschriftlichten Gespräche im Rahmen einer Zusammenfassung auf wesentliche Inhalte reduziert, um das Grundmaterial überblicken zu können. Im Anschluss wurde der Text je nach Bedeutung und Aussagekraft in einzelnen Sätzen oder Absätzen extrahiert, in dem zuvor die Analyseeinheiten bestimmt wurden (Sinnbereich, Interviewcode, Originaltext, Paraphrase, Generalisierung). Ehe die Kategorienbildung erfolgte, wurden die bedeutungstragenden Grundaussagen herausgearbeitet, anschließend weiter abstrahiert und zusammengefasst. Das erstellte Kategoriensystem orientierte sich eng am Leitfaden und wurde mit Fortgang der Auswertung wiederholt geprüft und ggf. modifiziert. Im Mittelpunkt stand dabei, die unterschiedlichen Erfahrungen, Sichtweisen und Bedürfnisse logisch zu kategorisieren. Als Reporting Statement wurde COREQ herangezogen.

## Ergebnisse

### Sample

Tab. 1 zeigt das gewonnene Sample.

**Table 1:** 

Alter	Ø 49 Jahre
Geschlecht	(31) weiblich, (6) männlich
Bildungsabschluss	(4) Volks‑/Hauptschulabschluss, (8) Mittlere Reife/Realschulabschluss, (16) (Fach‑)Abitur, (6) (Fach‑)Hochschulabschluss, (3) anderer Abschluss
Berufstätigkeit	(4) voll berufstätig, (17) teilweise berufstätig, (11) Rentner/in, (5) nicht berufstätig
Wohnumgebung	(15) groß- und mittelstädtisch, (22) kleinstädtisch-ländlich
Dauer der Pflegetätigkeit	(9) 6–12 Monate, (13) 1–3 Jahre, (10) 3–5 Jahre, (5) 5 Jahre und länger

### Pflegesettings

15 der 37 Interviewten übernehmen die Unterstützung bzw. Pflege allein, 22 teilen sich diese mit anderen Personen. In 19 Fällen hat die unterstützte Person einen Pflegegrad. In den meisten Fällen wird ein (Schwieger‑)Elternteil gepflegt (20), gefolgt vom Ehe- bzw. Lebenspartner (9), dem (Schwieger‑/Paten‑/Pflege‑)Kind (4) und sonstigen Verwandten (4). Die Hälfte der Interviewten (19) wohnt mit dem Gepflegten in einem Haushalt.

Der physische Zustand der unterstützten Person wird von 33 Interviewten als (sehr) stark eingeschränkt beschrieben; rund Zweidrittel (23) sprechen von (sehr) starken kognitiven Einschränkungen. Die Unterstützungstätigkeiten konzentrieren sich auf Betreuung und Beschäftigung im Alltag (36), Haushaltsführung bzw. -mithilfe (32), Unterstützung bei Körperpflege, Ernährung und Mobilität (29), Organisation von Hilfe und Pflege wie Antragstellungen, Behördengänge oder Arztbesuche (28). 17 Interviewte nennen darüber hinaus medizinisch-pflegerische Versorgungstätigkeiten.

Dreiviertel der Gesprächspartner*innen (27) beschreiben ihre persönliche Belastung bei der Pflege als (sehr) groß.

### Bedeutung der hausärztlichen Unterstützung

Eine deutliche Mehrheit der Interviewten erachtet den Hausarzt als prinzipiell wichtigen Ansprechpartner für Fragen der Pflege. 26 Personen geben an, mit dem eigenen oder einem anderen Hausarzt häufiger oder gelegentlich über die Pflegetätigkeit zu sprechen. Dabei machen viele der Gesprächspartner*innen deutlich, dass die persönliche Kenntnis und Vertrautheit des jeweiligen Hausarztes mit der eigenen Situation eine zentrale Voraussetzung ist, um in Fragen der Pflegetätigkeit Rat anzunehmen und Empfehlungen umzusetzen.

Das wäre der einzige medizinische Ratgeber, an den ich problemlos herankomme. Der meine Lage ganz einfach nachvollziehen und mir Tipps geben kann. […] Der Herr Doktor kennt mich und meine Familie. (I-17w)

Ich bin mir nicht sicher, ob die meisten Hausärzte sich selbst so sehen, dass sie in diesen Fragen eine Anlaufstelle sind. Aber aus unserer Sicht – also aus Sicht der pflegenden Leute – ist das ganz sicher so. (I-3m)

Das Gespräch über die pflegende Tätigkeit ist in den meisten Fällen dadurch zustande gekommen, dass die Angehörigen sich eigeninitiativ an ihren oder den Hausarzt des Gepflegten gewandt haben. Ein kleinerer Teil schildert, vom Hausarzt auf die Pflegesituation angesprochen worden zu sein oder dass sich im gemeinsamen Gespräch (z. B. Vorsorgeuntersuchung) das Thema „eher beiläufig ergeben“ habe (I-36w). Mehrere Interviewte drücken ihr Bedauern darüber aus, dass der Austausch über ihre pflegende Tätigkeit zunächst nicht stattgefunden habe, was sie u. a. darauf zurückführen, dass sie „ausgeprägte Unsicherheit“ verspürten (I-2m), ob es angemessen sei, über ihre private Pflegesituation zu sprechen und damit womöglich größere Zeitreserven zu beanspruchen.

Ehrlich gesagt war ich mir nicht sicher, ob ich jetzt wirklich das Recht habe, den Hausarzt mit diesen Problemen zu „nerven“. Da war ich eine Weile eher zurückhaltend […]. Erst als ich wirklich unter Druck stand mit meiner Situation zuhause habe ich mich überwunden. (I-11w)

Es macht schon einen Unterschied, ob Dir Signale gegeben werden, dass das Thema Pflege da in das Gespräch mit dem Arzt hineingehört oder nicht. Und diese Signale habe ich ganz einfach nicht bekommen […]. Das hat mich etwas zögern lassen. (I-7m)

Trotz solcher Verzögerungen, die sich teilweise ergaben, beschreiben die meisten Interviewten, die sich mit ihrem Hausarzt über ihre pflegende Tätigkeit austauschen, dass diese Gespräche (inzwischen) eher regelmäßig stattfinden. Während ein Teil der Befragten darstellt, dass es dem Hausarzt wichtig sei, „gesonderte Gespräche speziell zur Pflege“ (I-15m) zu führen, beschreibt ein anderer Teil, dieser Punkt werde bei Praxisbesuchen nach Bedarf mit behandelt.

### Betreuungsbedürfnisse pflegender Angehöriger

Im Hinblick auf Bedürfnisse und Erwartungen artikulieren die meisten Interviewten, dass sie sich jenseits eines ausreichendes Maßes an zur Verfügung gestellter Gesprächszeit und dem „Treffen von Entscheidungen im Einvernehmen“ (I-19w) ein „wirkliches Beschäftigen mit meiner persönlichen Pflegesituation“ (I-36w) wünschen, aber auch, dass der Hausarzt „sich für die Probleme von pflegenden Angehörigen zuständig fühlt“ (I-33m) und „nicht lange wartet, dass ich Probleme selbst anspreche“ (I-17w).

Bezogen auf die Personen, die mit dem Hausarzt über die Pflege sprechen, misst das Gros der Befragten diesem große Bedeutung im Sinne einer Informations- und Ratgeberquelle bei. Entsprechend wird von einem großen Teil des Samples der Wunsch artikuliert, dass der Hausarzt „früh auf Möglichkeiten zur weitergehenden Beratung und Hilfe hinweist“ (I-30w), „gerade was es hier in der nahen Umgebung so an Einrichtungen gibt, an die man sich wenden kann, um eine gute Pflege zu planen“ (I-33m). Einem größeren Teil genügen allgemeinere Übersichtsmöglichkeiten über beratende Einrichtungen (z. B. mittels Aushändigen von Broschüren), ein kleinerer Teil wünscht sich eine Vermittlung zu konkreten Hilfsangeboten.

Eine Gruppe der Interviewten spricht die Wichtigkeit rechtlicher Aspekte einer Pflege an und fände es günstig, wenn Hausärzt*innen hier Orientierungswissen vermitteln könnten. Ein vergleichbarer Anteil wünscht sich „so etwas wie emotionale Unterstützung“ (I-14w), „dass der Arzt einfach mal ein offenes Ohr für einen hat und einem zuredet, die guten Seiten an dieser manchmal vertrackten Pflegesituation zu sehen“ (I-22w).

### Zufriedenheit mit hausärztlicher Unterstützung

Von denjenigen Interviewten, die mit ihrem Hausarzt über ihre Pflegetätigkeit sprechen (26), zeigen sich die meisten (sehr) zufrieden mit der Art der hausärztlichen Unterstützung. Besonders positiv hervorgehoben wird das Vertrauensverhältnis beim Gespräch über die Pflegetätigkeit und die persönliche Kenntnis der häuslichen Pflegesituation. Zudem werden psychosoziale Aspekte der Unterstützung und Bestärkung genannt, die bei der Betreuung durch den Hausarzt als motivierend erlebt werden.

Es ist ein absoluter Vertrauensraum da, wo ich mich wohl fühle, anzusprechen, was mir auf der Seele brennt. […] Selbst wenn mir die Frau Ärztin nicht immer bei allem helfen kann, ist es gut zu wissen, dass sie mich kennt und Anteil nimmt. (I-33m)

Meine langjährige Hausärztin hat ein genaues Bild von meinem Alltag. Das spielt dann bei ihren Ratschlägen und Vorschlägen eine Rolle. Und natürlich ist das Vertrauen groß, auf sie zu hören. (I-27w)

Weiter wird von vielen Personen (20) positiv hervorgehoben, dass der Hausarzt auf den Gepflegten in gesundheitlicher wie mentaler Hinsicht eingehe (Bedürfnisse, Interessen, Entscheidungsfindung) und auf dessen Seite durch Erklärungen Einsicht schaffe.

Dennoch werden auch Schwachstellen der hausärztlichen Unterstützung angesprochen. So wurden Wünsche pflegender Angehöriger nach einer proaktiven hausärztlichen Rolle, z. B. bei zur rechtzeitigen Erfassung und Antizipation von Schwierigkeiten der Pflege, nicht immer erfüllt.

Es hat zu lange gedauert, bis sich da ein dauerhafter Gesprächskanal gebildet hat. Und das hat – obwohl ich und mein Angehöriger beim selben Hausarzt sind – einfach damit zu tun, dass der Hausarzt ein Gespräch über meine Pflege […] nicht gesucht hat. (I-19w)

Von verschiedenen Interviewten wird die Problematik angesprochen, dass Hausärzt*innen häufig ausschließlich auf den Gepflegten und dessen Situation fokussiert sind, ohne in gleichem Maße auf die Bedürfnisse und Belastungen der pflegenden Person einzugehen.

Unser Arzt sieht oft eigentlich nur meinen Mann, den ich pflege, ich hingegen bin zumeist ein wenig außen vor. Aber der Punkt ist ja: Das kann nur im Doppelpack funktionieren. Fühle mich manchmal schon etwas vernachlässigt. (I-28w)

Eine Reihe von Interviewten gibt an, bei mindestens einer Gelegenheit vom Hausarzt auf unterstützende Angebote aufmerksam gemacht worden zu sein, wobei am häufigsten auf Pflegedienste und Sozialstationen, Angebote zur Tages- oder Kurzzeitpflege und Angebote zur Alltagsunterstützung verwiesen wurde. Dies habe nach Einschätzung der Pflegenden ihre Arbeit längerfristig erleichtert. Allerdings hält ein großer Teil des Samples die hausärztliche Unterstützung in diesem konkreten Zusammenhang häufig für eher schwach ausgeprägt. Viele Angehörige wünschen sich eine stärker beratende Rolle des Hausarztes, wenn es um die Organisation von Rahmenbedingungen der Pflege geht.

Ich war schon etwas enttäuscht, dass mir da keine Unterstützungsmöglichkeiten genannt wurden […] und selbst auf Nachfrage kam wenig. (I-20m)

Ich musste mich dann mehr oder weniger allein informieren und mit Hilfe der Krankenkasse, was nicht so gut lief. (I-35w)

Mehrere Befragte bedauern zudem, von ihrem Hausärzt*innen nicht über rechtliche Aspekte beraten worden zu sein (v. a. Vorsorgevollmacht, Betreuung, „Autofahren“).

Tab. 2 fasst die wichtigsten Befunde, die sich im Zuge der Interviews ergeben haben, überblickartig zusammen.

**Table 2:** 

Wichtigkeit des Hausarztes als Ansprechpartner für Fragen der Pflege	(31) Sehr wichtig bzw. eher wichtig
(Regelmäßiges) Gespräch mit dem eigenen oder einem anderen Hausarzt über Pflegetätigkeit	(26)
Zustandekommen des (regelmäßigen) Gesprächs über Pflegetätigkeit	(17) Pflegende kommen mit Fragen bzw. Problemen auf Hausarzt zu; (9) initiativ von Hausarzt auf Pflege angesprochen
Wichtigste Betreuungsbedürfnisse	(25) Treffen von Entscheidungen im Einvernehmen; (25) Beschäftigung mit persönlicher Pflegesituation bzw. individuelles Eingehen auf selbige; (22) Hausarzt fühlt sich für Probleme Pflegender zuständig bzw. geht proaktiv auf Pflegende zu; (18) frühzeitiges Verweisen auf Informations- und Beratungsangebote; (9) Emotionale Unterstützung; (8) Vermittlung von rechtlichem Orientierungswissen
Hausarzt als Informations- und Ratgeberquelle	(25) Sehr wichtig bzw. eher wichtig
Zufriedenheit mit hausärztlicher Unterstützung insgesamt	(22) Sehr zufrieden bzw. eher zufrieden
Positiv erlebte Aspekte der hausärztlichen Unterstützung	(25) Vertrauensverhältnis; (25) persönliche Kenntnis der Pflegesituation; (22) psychosoziale Unterstützung bzw. Bestärkung; (20) therapeutische und psychosoziale Zuwendung zum Gepflegten
Negativ erlebte Aspekte der hausärztlichen Unterstützung	(14) keine rechtzeitige Ansprache auf Pflegesituation; (12) zaghaftes bzw. zu spätes Verweisen auf Hilfsangebote; (11) einseitige Fokussierung auf Gepflegten bzw. Vernachlässigung des Angehörigen

## Diskussion

### Zusammenfassung und Befunde anderer Studien

Die interviewten pflegenden Angehörigen erachten Hausärzt*innen als wichtige Unterstützungs- und Beratungsinstanz mit hoher Kompetenz- und Vertrauenszuweisung. Viele der Interviewten sprechen mit dem Hausarzt (regelmäßig) über ihre Pflegearbeit, wobei die hausärztliche Kenntnis der persönlichen Betreuungssituation, die Ansprechbarkeit bei verschiedensten Problemlagen und die Hinwendung zum Pflegebedürftigen besonders geschätzt wird. Demgegenüber fällt in einem Teil der Interviews auf, dass die Kommunikation über die Pflege oft erst deutlich verzögert erfolgt (verspätete Identifizierung und Ansprache Pflegender). Auch nehmen Hausärzt*innen nicht immer im selben Maße Rücksicht auf die Bedürfnisse von Angehörigen wie sie auf Gepflegte eingehen. Zudem deuten die Interviews darauf hin, dass nur ein Teil der Allgemeinärzt*innen bei der Unterstützung von Angehörigen auf Beratungs- und Hilfsangebote verweist.

Die generellen Befunde der vorliegenden Studie refelektieren damit nicht nur zentrale Befunde der vorangegangenen Online-Befragung [27], sondern auch andere Erhebungen, die die Bedeutung der Hausarztpraxis für pflegende Personen betonen [7, 14, 23, 24, 29]. Zudem existiert eine Reihe internationaler qualitativer Arbeiten, die sich mit der Perspektive Pflegender befasst haben und deren Befunde Ähnlichkeiten mit der durchgeführten Studie aufweisen. So hebt eine in Irland realisierte Interviewstudie mit pflegenden Personen die prioritäre Rolle des Hausarztes hervor, wenn es darum geht, längerfristige Coping- und Resilienzstrategien in häuslichen Pflegesettings zu entwickeln [30]. Auch zeigte sich hier der Bedarf nach einer kontinuierlichen, proaktiven Betreuung durch einen festen ärztlichen Ansprechpartner, ein tiefergehendes Verständnis von privater Pflege sowie die Erfordernis passgenauer Versorgungslösungen. Greenwood und Kolleg*innen [26] konnten ihrerseits in einer qualitativen Studie herausarbeiten, dass das primärärztliche Setting eine zentrale Versorgungsaufgabe bei der Unterstützung spezifischer Gruppen von pflegenden Personen wahrnehmen und die weitere Versorgung effektiv und kollaborativ koordinieren kann.

Wie auch die vorliegende Arbeit gezeigt hat, lassen sich in anderen durchgeführten Studien zum Thema Schwachpunkte der hausärztlichen Versorgung einkreisen. So fällt in den qualitativen Untersuchungen von Burridge et al. auf, dass Pflegende sich nicht immer trauen, ihre Problematiken darzulegen, wenn Hausärzt*innen ihnen nicht signalisieren, dass sie sich als Anlaufstelle verstehen [25, 31]. Damit pflegende Angehörige ihre Anliegen und Probleme artikulieren, ist es wichtig, ihnen frühzeitig zu signalisieren, dass ihre Unterstützung in den hausärztlichen Zuständigkeitsbereich fällt. Anlässe wie Gesundheits-Check-ups oder Impfungen bieten sich an, um initiativ auf pflegende Personen zuzugehen. Auch kommt es vor, dass Allgemeinärzt*innen nicht immer im selben Maße Rücksicht auf die Bedürfnisse von Angehörigen nehmen wie sie auf pflegebedürftige Patient*innen eingehen. Die Forschungsliteratur spricht hier die Tendenz an, pflegende Angehörige vorrangig in Bezug auf die gepflegte Person wahrzunehmen, wobei psychosoziale Auswirkungen marginalisiert werden [21, 25, 26]. Wie verschiedene Arbeiten betonen, ist zur effektiven Unterstützung einer gelingenden Pflege bedeutend, in der triadischen Konstellation die Bedürfnisse, Wünsche und Belastungen Pflegender und Gepflegter gleichermaßen zu berücksichtigen [24–26, 29, 32].

Zudem zeigen Arbeiten wie jene von Kiceniuk et al., dass der Bedarf pflegender Personen und Gepflegter nach frühzeitigen, konsequenten und systematischen Verweisen auf Beratungs- und Hilfsangebote nicht immer von Hausärzten adressiert wird. Indem pflegende Angehörige an solche Unterstützungsakteure vermittelt werden, erhalten sie rechtzeitigen Zugang zu Informationen zur Organisation der Pflege [8, 29], die einen längeren Aufenthalt der Pflegepersonen zuhause ohne Versorgungskrisen (z. B. Hospitalisierungen) ermöglichen [19, 33].

Die nicht immer gegebene Vermittlungstätigkeit von Hausärzten hin zu Hilfsnetzen korrespondiert mit dem Befund, dass Hausärzte oft keinen ausreichenden Überblick über externe Unterstützungsformen für pflegende Personen haben [19, 24] und selten in kommunale Gesundheitsnetzwerke eingebunden sind [34, 35]. Eine weitere Schwierigkeit besteht darin, dass das hausärztliche Team pflegende Angehörige nicht immer zeitnah identifiziert [36]. Die genannten Herausforderungen sind bereits im Zuge der Online-Befragung aufgefallen. Solche Problematiken könnten damit zusammenhängen, dass – wie Bedard et al. aufzeigen – Hausärzte zum einen oft nicht die Zeit und Ressourcen zur Verfügung stellen, um pflegenden Angehörigen vollumfänglich gerehcht zu werden [37]. Zudem konnten Carduff et al. eruieren, dass Hausärzte oft ein stark unterschiedliches Rollenverständnis in Bezug auf ihren Umgang mit Pflegenden vertreten [38].

### Stärken und Schwächen

Die vorliegende Studie ermöglichte es, ein heterogenes Sample pflegender Personen in Bezug auf ihre Betreuungsbedürfnisse im hausärztlichen Setting zu befragen. Sie weist eine Reihe von Limitationen auf, die entsprechend zu reflektieren sind.

So wurde kein separater Pretest durchgeführt. Zudem handelt es sich um eine nicht-repräsentative Studie mit kleinem Sample, das nicht stellvertretend für die Gesamtheit pflegender Angehöriger ist. Die Rekrutierung über Online-Foren macht es insgesamt wahrscheinlich, dass eine bestimmte Gruppe mit spezifischen Informations- und Austauschbedürfnissen für das Sample gewonnen werden konnte. Entsprechend ist davon auszugehen, dass die Rekrutierung von pflegenden Personen in anderen Settings (z. B. Wartezimmerbefragungen in hausärztlichen Praxen) zu möglicherweise besser verallgemeinerbaren Aussagen über die Grundgesamtheit pflegender Angehöriger führen würde. Ferner ist anzumerken, dass die telefonischen Interviews zu einer geringeren Auskunftsbereitschaft als im Fall von Face-to-face-Interviews geführt haben könnten.

In Bezug auf den Leitfaden muss eingewandt werden, dass dieser auch eine Reihe quantitativer Einschätzungsfragen enthielt, in denen der Spielraum für persönliche Ausführungen und Interpretation klar begrenzt war. Insofern ist die Studie in Bezug auf ihren Charakter als qualitative Arbeit unter besagten Einschränkungen zu betrachten. Narrative Interviews können pflegenden Angehörigen ausgedehntere Möglichkeiten eröffnen, ihren Standpunkt, ihre Erfahrungen und Befindlichkeiten im Erzähl- und Erklärungsfluss darzulegen.

## Schlussfolgerungen

Im Zuge der durchgeführten Interviews hat sich gezeigt, dass die Hausarztpraxis eine vitale Rolle bei der Unterstützung pflegender Angehöriger einnehmen und diesen Bestärkung und Sicherheit im Hinblick auf die Pflegeorganisation vermitteln kann. Ein großer Teil der Interviewten tauscht sich regelmäßig mit dem Hausarzt über die Pflege aus, wobei v. a. die hausärztliche Kenntnis der persönlichen Betreuungssituation, die Ansprechbarkeit bei verschiedensten Problemlagen und die Hinwendung zum Pflegebedürftigen als positiv erlebt wird.

Indem Hausärzt*innen auf die Bedürfnisse Pflegender eingehen, sind sie in der Lage, häusliche Pflegesettings längerfristig zu stabilisieren sowie einem „Ausbrennen“ der Pflegeperson vorzubeugen. Dazu sollten pflegende Angehörige früh identifiziert und angesprochen werden. Zudem ist es wichtig, in der triadischen Konstellation die Bedürfnisse, Wünsche und Belastungen Pflegender und Gepflegter gleichermaßen zu berücksichtigen.

Ein zentraler Hebel für die Effektivierung der hausärztlichen Unterstützung pflegender Angehöriger besteht in einer besseren und systematischeren Verzahnung von Hausärzten mit Beratungs- und Unterstützungsakteuren wie z. B. Pflegestützpunkten, ambulanten psychiatrischen Diensten und Demenz-Netzwerken. Indem Patient*innen und Angehörige rechtzeitig an derlei Hilfsnetzwerke herangeführt werden, wird die Aufrechterhaltung einer guten Lebensqualität für beide entscheidend unterstützt. Hierfür wird es darauf ankommen, die interdisziplinäre sektorenübergreifende Kommunikation zu stärken, (in)formelle Kooperationsnetze zu errichten und Hausärzt*innen eine belastbare Kenntnis von beratenden Akteuren in ihrer Umgebung zu vermitteln.

### Supplementary Information





## References

[1] Eurostat. Bevölkerungsstruktur und Bevölkerungsalterung. 2021. https://ec.europa.eu/eurostat/statistics-explained/index.php?title=Population_structure_and_ageing/de. Zugegriffen: 1. Juni 2021.

[2] WHO Regional Office for Europe (2015). Home care in Europe.

[3] Statistisches Bundesamt. Pflegestatistik 2019. 2020. https://www.destatis.de/DE/Themen/Gesellschaft-Umwelt/Gesundheit/Pflege/Publikationen/_publikationen-innen-pflegestatistik-deutschland-ergebnisse.html. Zugegriffen: 1. Juni 2021.

[4] Nowossadeck S, Engstler H, Klaus D (2016). Pflege und Unterstützung durch Angehörige.

[5] Tesch-Römer C, Hagen C. Ausgewählte Aspekte zur informellen häuslichen Pflege in Deutschland. 2018. https://nbn-resolving.org/urn:nbn:de:0168-ssoar-58856-1. Zugegriffen: 1. Juni 2021.

[6] Connell CM, Boise L, Stuckey JC (2004). Attitudes toward the diagnosis and disclosure of dementia among family caregivers and primary care physicians. Gerontologist.

[7] DAK (2015). DAK-Pflege-Report 2015.

[8] Wuttke-Linnemann A, Henrici CB, Müller N (2020). Bouncing back from the burden of dementia: predictors of resilience from the perspective of the patient, the spousal caregiver, and the dyad—an exploratory study. GeroPsych.

[9] Klaus D, Tesch-Römer C, Mahne K, Wolff J, Simonson J (2016). Pflege und Unterstützung bei gesundheitlichen Einschränkungen: Welchen Beitrag leisten Personen in der zweiten Lebenshälfte für andere?. Altern im Wandel: Zwei Jahrzehnte Deutscher Alterssurvey.

[10] Schmidt M, Schneekloth U (2011). Abschlussbericht zur Studie „Wirkungen des Pflege-Weiterentwicklungsgesetzes“.

[11] Bestmann B, Wüstholz E, Verheyen F (2014). Belastung und sozialer Zusammenhalt. Eine Befragung zur Situation von pflegenden Angehörigen.

[12] O’Reilly D, Connolly S, Rosato M (2008). Is caring associated with an increased risk of mortality? A longitudinal study. Soc Sci Med.

[13] Beach SR, Schulz R, Williamson GM (2005). Risk factors for potentially harmful informal caregiver behavior. J Am Geriatr Soc.

[14] Cherry MG, Salmon P, Dickson JM (2013). Factors influencing the resilience of carers of individuals with dementia. Rev Clin Gerontol.

[15] Schulz R, Sherwood P (2008). Physical and mental health effects of family caregiving. Am J Nurs.

[16] Dias R, Santos RL, Sousa MF (2015). Resilience of caregivers of people with dementia: a systematic review of biological and psychosocial determinants. Trends Psychiatry Psychother.

[17] Joling K, Windle G, Dröes R-M (2016). Factors of resilience in informal caregivers of people with dementia from integrative international data analysis. Dement Geriatr Cogn Disord.

[18] Roepke SK, Mausbach BT, Patterson TL (2011). Effects of Alzheimer caregiving on allostatic load. J Health Psychol.

[19] Geschke K, Scheurich A, Schermuly I (2012). Effectivity of early psychosocial counselling for family caregivers in general practitioner based dementia care. Dtsch Med Wochenschr.

[20] Romero-Moreno R, Márquez-González M, Mausbach BT (2012). Variables modulating depression in dementia caregivers: a longitudinal study. Int Psychogeriatr.

[21] Bulsara CE, Fynn N (2006). An exploratory study of gp awareness of carer emotional needs in Western Australia. BMC Fam Pract.

[22] Greenwood N, Mackenzie A, Habibi R (2010). General practitioners and carers: a questionnaire survey of attitudes, awareness of issues, barriers and enablers to provision of services. BMC Fam Pract.

[23] Kassenärztliche Bundesvereinigung (2018). Versichertenbefragung 2018. Ergebnisse einer repräsentativen Bevölkerungsumfrage.

[24] Laux N, Melchinger H, Scheurich A (2010). Improving general practitioners guided dementia care. Dtsch Med Wochenschr.

[25] Burridge LH, Mitchell GK, Jiwa M (2011). Consultation etiquette in general practice: a qualitative study of what makes it different for lay cancer caregivers. BMC Fam Pract.

[26] Greenwood N, Mackenzie A, Harris R (2011). Perception of the role of general practice and practical support measures for carers of stroke survivors: a qualitative study. BMC Fam Pract.

[27] Wangler J, Jansky M (2021). Support, needs and expectations of family caregivers regarding general practitioners—results from an online survey. BMC Fam Pract.

[28] Mayring P (2010). Qualitative Inhaltsanalyse. Grundlagen und Techniken.

[29] Donath C, Gräßel E, Großfeld-Schmitz M (2010). Effects of general practitioner training and family support services on the care of home-dwelling dementia patients—results of a controlled cluster-randomized study. BMC Health Serv Res.

[30] Lane P, McKenna H, Ryan A (2003). The experience of the family caregivers’ role: a qualitative study. Res Theory Nurs Pract.

[31] Burridge LH, Mitchell G, Jiwa M (2017). Helping lay carers of people with advanced cancer and their GPs to talk: an exploration of Australian users’ views of a simple carer health checklist. Health Soc Care Community.

[32] The Princess Royal Trust for Carers and Royal College of General Practitioners (2011). Supporting carers: an action guide for general practitioners and their teams.

[33] Thyrian JR, Fiss T, Dreier A (2012). Life- and person-centred help in Mecklenburg-Western Pomerania, Germany (DelpHi): study protocol for a randomised controlled trial. Trials.

[34] Kuske S, Graf R, Hartig M (2014). Dementia considered? Safety-relevant communication between health care settings: a systematic review. J Public Health.

[35] Prüfer F, Joos S, Milksch A (2015). Die Rolle des Hausarztes in der kommunalen Gesundheitsförderung. Präv Gesundheitsf.

[36] Wangler J, Fellgiebel A, Jansky M (2018). Dementia diagnosis in general practitioner care—attitudes, procedures and challenges from the perspective of general practitioners in Rhineland-Palatinate. Dtsch Med Wochenschr.

[37] Bedard M, Gibbons C, Lambert-Belanger A (2014). Development of a tool to investigate caregiving issues from the perspective of family physicians and discussion of preliminary results. Prim Health Care Res Dev.

[38] Carduff E, Finucane A, Kendall M (2014). Understanding the barriers to identifying carers of people with advanced illness in primary care: triangulating three data sources. BMC Fam Pract.

