# Supplementary material for: What prevention potential does the general practitioner setting offer for family caregivers?—findings from a qualitative interview study
Source: Wien Med Wochenschr. 2021 Sep 16;174(3-4):35–43. [Article in German] doi: 10.1007/s10354-021-00880-4 (PMC10896783; doi:10.1007/s10354-021-00880-4)
Supplement: Supplementary file 1 [file 10354_2021_880_MOESM1_ESM.docx]

**Interviewleitfaden**

*Formale Fragen zum Pflegesetting*

**Übernehmen Sie die Unterstützung bzw. Pflege allein oder teilen Sie diese mit anderen Personen?** (Pflegedienste oder Sozialstationen, z.B. im Rahmen der Pflegeversicherung, sind nicht gemeint.)

**Hat die von Ihnen unterstützte Person einen Pflegegrad?**

**Wie lange helfen Sie dieser Person schon?**

**In welchem Verhältnis steht die durch Sie unterstützte Person zu Ihnen?**

**Was würden Sie sagen: Wie stark ist die von Ihnen unterstützte Person eingeschränkt? Worauf beziehen sich die Einschränkungen vor allem?**

**Wohnen Sie und die von Ihnen unterstützte Person aktuell im selben Haushalt?**

**In welcher Weise unterstützen Sie Ihre/n Angehörige?**

**Als wie groß erleben Sie die Belastung bei der Pflege der von Ihnen unterstützten Person?**

*Fragen zur hausärztlichen Unterstützung*

**Wie wichtig ist Ihnen der/die Hausärzt/in als Ansprechpartner/in für Fragen zur Pflege Ihres Angehörigen?**

**Sprechen Sie mit Ihrem oder einem/einer anderen Hausärzt/in über die Pflege Ihres/Ihrer Angehörigen?** (Gemeint ist nicht unbedingt Ihr/e eigene/r Hausärzt/in, sondern derjenige/diejenige Hausärzt/in, mit dem/der Sie über die Pflege Ihres/Ihrer Angehörigen sprechen.)

**Wie ist das Gespräch über die Pflege Ihres/Ihrer Angehörigen zustande gekommen?**

**Wie häufig sprechen Sie mit Ihrem/Ihrer oder einem/einer anderen Hausärzt/in über die Pflege des/der von Ihnen unterstützten Angehörigen? Wie regelmäßig finden diese Gespräche statt?**

**Pflegende Angehörige können ganz unterschiedliche Erwartungen und Bedürfnisse haben, wie der/die Hausärzt/in sie unterstützen sollte. Was ist Ihnen als pflegende/r Angehörige/r wichtig, wenn Sie speziell an den/die Hausärzt/in denken? Was wünschen Sie sich vor allem?**

**Gerade haben wir von Ihren Wünschen und Erwartungen gesprochen. Wie erleben Sie in der Rolle als pflegende/r Angehörige/r tatsächlich die hausärztliche Unterstützung? Welche Art der Unterstützung haben Sie von hausärztlicher Seite erhalten? Was läuft gut, womit sind Sie zufrieden? Und wo würden Sie sich mehr Unterstützung durch den/die Hausärzt/in wünschen, womit sind Sie ggf. unzufrieden?**

**Was würden Sie sich, insgesamt betrachtet, stärker vom Hausärzt/in in Bezug auf die Unterstützung bei der Pflege wünschen?**

**Neulich sagte jemand: „Wenn ich mit einer Frage zur Pflege meines/meiner Angehörigen auf meine/n Hausärzt/in zukomme, dann kann diese/r mir für gewöhnlich gut weiterhelfen.“ Wie stark trifft das auf Ihre Hausärzt/in zu? Warum (nicht)?**

**Welche Bedeutung hat der/die Hausärzt/in für Sie, wenn es speziell darum geht, Informationen zur Pflege zu bekommen, also z.B. welche Dienste es gibt oder an wen Sie sich zur weiteren Beratung wenden können?**

**Wie häufig hat Sie der/die Hausärzt/in auf unterstützende Hilfs- oder Beratungsangebote zur Organisation der Pflege aufmerksam gemacht?** (Gemeint ist nicht unbedingt, dass der/die Ärzt/in auf konkrete Angebote vor Ort hinweist, sondern Sie generell auf solche Unterstützungsmöglichkeiten angesprochen hat.) **Auf welche Angebote hat der/die Hausärzt/in Sie aufmerksam gemacht?**

**Haben Sie diese(s) Beratungsangebot(e) wahrgenommen? Wie viel hat es gebracht bzw. inwiefern hat es Ihre Pflegetätigkeit erleichtert? / Glauben Sie, es hätte Ihnen etwas gebracht, wenn der/die Hausärzt/in Sie zu konkreten Hilfs- und Betreuungsangeboten vermittelt hätte?**

**Versuchen Sie eine Bilanz zu ziehen: Wie gut fühlen Sie sich durch Ihre/n Hausärzt/in bei der Pflege Ihres/Ihrer Angehörigen insgesamt unterstützt?**

**Ist es schon einmal vorgekommen, dass Sie den Hausarzt gewechselt haben, weil Sie unzufrieden mit dessen Unterstützung bei der Pflege Ihres/Ihrer Angehörigen waren?**

**Was sollte oder müsste Ihrer Meinung getan oder verbessert werden, damit pflegende Angehörige besser unterstützt werden und auf Ihre Bedürfnisse und Probleme eingegangen werden kann? Wie kann speziell der Hausarzt (besser) dabei helfen?**
